# Lobophorin K, a New Natural Product with Cytotoxic Activity Produced by *Streptomyces* sp. M-207 Associated with the Deep-Sea Coral *Lophelia pertusa*

**DOI:** 10.3390/md15050144

**Published:** 2017-05-19

**Authors:** Alfredo F. Braña, Aida Sarmiento-Vizcaíno, Miguel Osset, Ignacio Pérez-Victoria, Jesús Martín, Nuria de Pedro, Mercedes de la Cruz, Caridad Díaz, Francisca Vicente, Fernando Reyes, Luis A. García, Gloria Blanco

**Affiliations:** 1 Departamento de Biología Funcional, Área de Microbiología, Instituto Universitario de Oncología del Principado de Asturias, Universidad de Oviedo, 33006 Oviedo, Spain; afb@uniovi.es (A.F.B.); UO209983@uniovi.es (A.S.-V.); UO21622@uniovi.es (M.O.); 2 Fundación MEDINA, Centro de Excelencia en Investigación de Medicamentos Innovadores en Andalucía, Avda. del Conocimiento 3, Parque Tecnológico de Ciencias de la Salud, E-18016 Granada, Spain; ignacio.perez-victoria@medinaandalucia.es (I.P.-V.); jesus.martin@medinaandalucia.es (J.M.); nuria.de.pedro@medinaandalucia.es (N.d.P.); mercedes.delacruz@medinaandalucia.es (M.d.l.C.); caridad.diaz@medinaandalucia.es (C.D.); francisca.vicente@medinaandalucia.es (F.V.); 3 Departamento de Ingeniería Química y Tecnología del Medio Ambiente, Área de Ingeniería Química, Universidad de Oviedo, 33006 Oviedo, Spain; luisag@uniovi.es

**Keywords:** lobophorins, spirotetronate antibiotic, *Streptomyces*, antitumor, Cantabrian Sea-derived actinobacteria

## Abstract

The present article describes the isolation of a new natural product of the lobophorin family, designated as lobophorin K (**1**), from cultures of the marine actinobacteria *Streptomyces* sp. M-207, previously isolated from the cold-water coral *Lophelia pertusa* collected at 1800 m depth during an expedition to the submarine Avilés Canyon. Its structure was determined using a combination of spectroscopic techniques, mainly ESI-TOF MS and 1D and 2D NMR. This new natural product displayed cytotoxic activity against two human tumor cell lines, such as pancreatic carcinoma (MiaPaca-2) and breast adenocarcinoma (MCF-7). Lobophorin K also displayed moderate and selective antibiotic activity against pathogenic Gram-positive bacteria such as *Staphylococcus aureus*.

## 1. Introduction

Lobophorins are natural products of marine origin with pharmacological interest due to their diverse biological activities. Structurally, they are members of the spirotetronate family first reported as anti-inflammatory compounds, such as lobophorins A and B [1]. Antibiotic activities were subsequently reported for other members of the family, such as lobophorins E and F [2], lobophorin G [3], and lobophorins H and I [4,5]. Unfortunately, due to an overlapping in the publication dates, the two last references add some confusion to the nomenclature of the lobophorin family, as the names lobophorin H and I were assigned twice to new compounds whose structures are not coincident, isolated independently by both research groups. The compound named lobophorin I in the Lin paper [5] has the same structure as lobophorin H in the Pan article [4], and the compound whose name was assigned as lobophorin H in the Lin paper would represent a new natural product whose name should be perhaps be reassigned as lobophorin I* in order to not add more confusion to the nomenclature of these molecules, taking into account the publication date of both articles. Very recently, three additional members of the lobophorin family—namely lobophorins CR1, CR2, and CR3 were isolated from cultures of *Streptomyces* sp. strain 7790N4 [6]. Finally, the isolation of a compound named lobophorin J from a culture of the deep sea-derived *Streptomyces* sp. strain 12A35 has also been reported [7]. In addition to their antimicrobial activities, some lobophorins also display cytototoxic activity against different tumor cell lines, such as lobophorins C and D against liver and breast tumor cell lines [8], lobophorin F against SNC, breast, and lung tumor cell lines [2], and lobophorins CR1 and CR2 against oral cancer cell lines [6].

Previous research in the Cantabrian Sea (Biscay Bay), Northeast Atlantic, has revealed that bioactive actinobacteria, mainly *Streptomyces* species, are associated to corals and other invertebrates living up to 4700 m depth in the submarine Avilés Canyon [9,10,11]. Recently, actinobacteria displaying a wide repertoire of chemically diverse secondary metabolites with different antibiotic or antitumor activities have been isolated from coral reef ecosystems from the Avilés Canyon [12]. We report here the finding of a new natural product, lobophorin K, obtained from *Streptomyces* sp. M-207, isolated from the cold-water coral *Lophelia pertusa* collected at 1800 m depth in this submarine canyon. Following our LC-UV-MS and LC-HRMS chemical dereplication approaches for marine natural products [13], it was determined that the extract obtained from fermentation broths of this strain contained lobophorins A and B, together with a new member of the lobophorin family. After targeted isolation, we have shown that this new compound, lobophorin K, displays cytotoxic activities against two human tumor cell lines, particularly pancreatic carcinoma and breast adenocarcinoma and it also presents moderate and selective antibiotic activity against pathogenic Gram-positive bacteria such as *S. aureus*.

## 2. Results

### 2.1. Taxonomy and Phylogenetic Analysis of the Strain M-207

**Figure 1:**
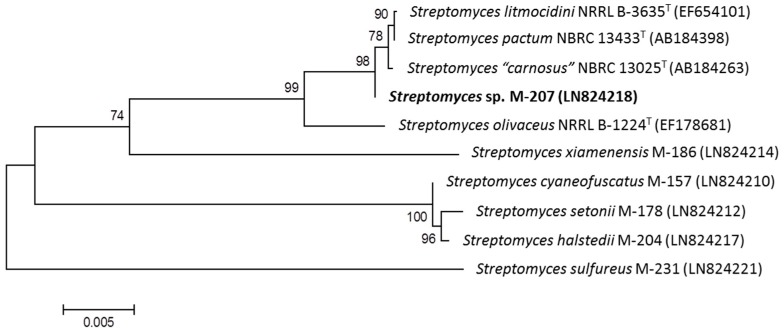
Neighbor-joining tree based on 16 S rDNA sequence of strain M-207 and the closest strains of the genus *Streptomyces*.

The 16S rDNA of producing strain M-207 was amplified by polymerase chain reaction (PCR) and sequenced [12]. After Basic Logic Alignment Search Tool (BLAST) sequence comparison strain M-207 showed 99.89% (920/921) identities to *Streptomyces carnosus* NBRC 13025T, *Streptomyces olivaceus* NRRL B-3009T, and *Stresptomyces pactum* NBRC 13433T. The phylogenetic tree generated by a neighbor-joining method based on 16S rDNA sequence clearly revealed the evolutionary relationship of the strain M-207 with these three known *Streptomyces* species (Figure 1). So, in the absence of further taxonomic analysis the strain was designated as *Streptomyces* sp. M-207.

### 2.2. Structure Determination

**Figure 2:**
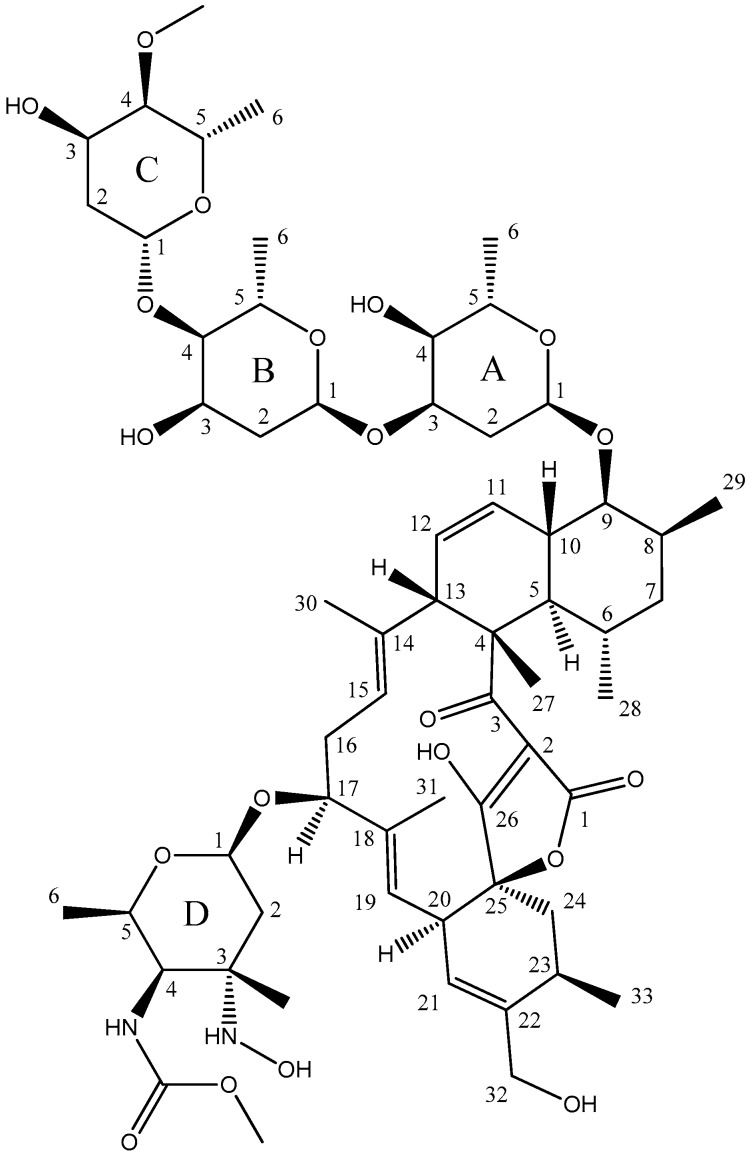
Chemical structure of lobophorin K (**1**).

Compound **1** had a molecular formula of C_61_H_92_N_2_O_20_ according to the protonated ion at *m*/*z* 1173.6322 observed in its ESI-TOF spectrum (calc. for C_61_H_93_N_2_O_20_^+^, 1173.6316, ∆ 0.5 ppm). Signals observed in its ^1^H and ^13^C NMR spectra (Table 1) and correlations observed in the 2D NMR spectra were indicative of a lobophorin-like structure for the compound. The molecule had an additional oxygen atom compared with lobophorin A, and the major differences between the spectra of this new compound and those of this molecule and other lobophorins were found around the deoxyaminosugar moiety attached to carbon C17 (unit D). In most of the compounds of the lobophorin series, an amino (–NH_2_) or a nitro (–NO_2_) substituent is found at position D3. Interestingly, the new lobophorin bears a hydroxylamino (–NHOH) substituent at that position. This indeed represents an intermediate oxidation state between the amino and nitro functional groups found in the lobophorin series at position D3. Such an intermediate oxidation state would represent and intermediate biosynthetic product among both types of lobophorins. For lobophorin H and lobophorin I (according to [5]), carrying respectively amino and nitro substitution at position D3, NMR data have been reported in CD_3_OD, allowing a direct comparison of the chemical shifts observed for ring D with those of the new lobophorin herein reported. Such comparison revealed the expected differences, especially in the resonance frequency of carbon at position D3, which is found at 61.6 ppm in the new lobophorin and at 57.0 and 92.9 ppm in lobophorins H and I respectively. The carbon chemical shift measured at position D3 for the new lobophorin is in agreement with the expected deshielding for the hydroxylamino functionality (61.6 ppm) with respect to the amino functionality (57.0 ppm), though it is strongly shielded compared to the nitro functionality (61.6 ppm vs. 92.9 ppm). Compound **1** therefore had the structure depicted in Figure 2, being the hydroxylamino derivative of lobophorin A.

### 2.3. Antimicrobial Activity of Lobophorin K

In order to determine the antibiotic properties of the molecule, it was tested against a panel of Gram-positive and Gram-negative clinical pathogens. As shown in Table 2, it did not exhibit remarkable antibacterial activities against Gram-negative bacteria such as *Pseudomonas aeruginosa*, *Acinetobacter baumannii*, *Klebsiella pneumoniae*, or *Escherichia coli*. However, lobophorin K displayed moderate and selective activity against Gram-positive bacteria such as methicillin sensitive *S. aureus* EPI167, with a MIC_90_ value of 40–80 µg/mL.

### 2.4. Cytotoxic Activity of Lobophorin K

The compound showed cytotoxic activity with IC_50_ values of 23.0 ± 8.9, 34.0 ± 85.1, and 6.3 ± 8.2 µM against a human breast adenocarcinoma cell line (MCF-7), a human pancreatic carcinoma cell line (MiaPaca-2), and a human immortalized hepatocyte cell line (THLE-2), respectively (Figure S9). Lobophorin K was inactive at the maximum concentration tested (42.6 µM) against other human tumor cell lines such as lung (A-549), colon (HT-29), and hepatocarcinoma (HepG2).

## 3. Experimental Section

### 3.1. General Experimental Procedures

All solvents were purchased from VWR Chemicals (Barcelona, Spain). Analytical and semi-preparative HPLC was conducted using a Waters Alliance chromatographic system (Waters Corporation, Mildford, MA, USA) with a SunFire C18 column (10 µm, 10 × 250 mm, Waters). For UPLC analysis, an Acquity UPLC equipment (Waters) with a BEH C18 column (1.7 μm, 2.1 × 100 mm, Waters) was used. Optical rotations were determined with a JASCO P-2000 polarimeter (JASCO Corporation, Tokyo, Japan). IR spectrum was measured with a JASCO FT/IR-4100 spectrometer (JASCO Corporation) equipped with a PIKE MIRacle^TM^ single reflection ATR accessory. NMR spectra were recorded on a Bruker Advance III spectrometer (500 and 125 MHz for ^1^H and ^13^C NMR, respectively) equipped with a 1.7 mm TCI MicroCryoProbe^TM^ (Bruker Biospin, Fällanden, Switzerland), using the signal of the residual solvent as internal reference (δ_H_ 3.31 and δ_C_ 49.0 ppm for CD_3_OD). ESI-TOF MS spectra were acquired using a Bruker maXis QTOF spectrometer (Bruker Daltonik GmbH, Bremen, Germany).

### 3.2. Phylogenetic Analysis of the Producing Microorganism

Strain *Streptomyces* sp. M-207 was subjected to phylogenetic analysis based on 16S rDNA sequence analysis [12]. Phylogenetic analysis was performed using MEGA version 6.0 [14] after multiple alignment of data by Clustal Omega [15]. Distances were calculated using distance options according to the Kimura two-parameter model [16]. Clusters constricted by the neighbor-joining method [17] were evaluated using bootstrap values based on 1000 replications [18].

### 3.3. Microorganism and Fermentation Conditions

Strain M-207 was isolated from a deep-sea coral sample collected from the Cantabrian Sea at the depth of 1800 m [12]. 30 Erlenmeyer flasks (250 mL), each containing 50 mL of R5A medium, as previously described [9] were inoculated with spores and incubated in an orbital shaker at 28 °C and 250 rpm for four days.

### 3.4. Isolation and Purification of Lobophorin K

The cultures were centrifuged and pellets and supernatants were processed separately. The pellets were extracted twice with ethyl acetate acidified with 1% formic acid. The supernatants were filtered and applied to a solid-phase extraction cartridge (Sep-Pak Vac C18, 10 g, Waters). The retained material was eluted with a mixture of methanol and 0.05% TFA in water. A linear gradient from 0 to 100% methanol in 60 min, at 10 mL/min, was used. Fractions were taken every 5 min and analyzed by UPLC using chromatographic conditions previously described [19]. A peak corresponding to the unknown lobophorin was observed in fractions taken between 45 and 55 min. These fractions were pooled, dried in vacuo, and the residue was subsequently re-dissolved in a small volume of DMSO and methanol (1:1). The same peak was also found in the organic extract of the culture pellets, which was processed in the same way. The desired compound was purified by semi-preparative HPLC using a SunFire C18 column (10 µm, 10 × 250 mm, Waters). The mobile phase was a mixture of acetonitrile and 0.05% TFA in water in isocratic conditions, at 7 mL/min. The purification was performed in two steps, with 90% acetonitrile in the first step and 50% in the second step. In both cases, the solution containing the collected peak was partially evaporated under vacuum to reduce the concentration of the organic solvent and then applied to a solid-phase extraction cartridge (Sep-Pak C18, Waters). The cartridge was washed with water, the retained compound was eluted with methanol and finally lyophilized, resulting in 1.9 mg of pure compound **1**.

Lobophorin K (**1**): yellowish amorphous solid; ${\left[\alpha \right]}_{D}^{20}$ −102.2 (*c* 0.1, MeOH); IR (ATR) υ_max_ 3427, 2931, 1708, 1630, 1549, 1454, 1412, 1378, 1126, 1095, 1060, 1013, 926, 868 cm^−1^; for ^1^H and ^13^C NMR data see Table 1; HRESIMS *m/z* 1173.6322 [M + H]^+^ (calcd. for C_61_H_93_N_2_O_20_^+^, 1173.6316), 1195.6141 [M + Na]^+^ (calcd. for C_61_H_92_N_2_O_20_Na^+^, 1195.6136).

### 3.5. Antimicrobial Activity of Lobophorin K

Previously described methods, using pathogenic strains from Fundación MEDINA’s collection were used to test for antibiotic properties [20]. Briefly, the compound was serially diluted in DMSO with a dilution factor of 2 to provide 10 concentrations for all the assays. The MIC was defined as the lowest concentration of compound that inhibited ≥90% of the growth of a microorganism after overnight incubation. Genedata Screener software (Genedata, Inc., Basel, Switzerland) was used to analyze the data and to calculate the RZ’ factor to estimate the robustness of the assays [21]. In all experiments performed in this work, the RZ’ factor obtained was between 0.85 and 0.92. The compound was tested in two different days and in triplicate at final assay concentrations ranging between 160 and 0.31 µg/mL.

### 3.6. Cytotoxic Activity of Lobophorin K

Five tumor cell lines (MCF-7, MiaPaca-2, HepG2, A-549, and HT-29) and one non-tumor cell line (THLE-2) were obtained from ATCC. The MTT (3-(4,5-dimethylthiazol-2-yl)-2,5-diphenyltetrazolium bromide) colorimetric assay, which measures mitochondrial metabolic activity, was employed to estimate the amount of living cells. Ten thousand cells per well (for 72 h assay) were plated in 96-well plates with a cell culture robotic system, SelecT (TAP Biosystems, Royston, UK). After 24 h of incubation, lobophorin K and solvent as blank were added with the automated liquid-handling system Biomek FX (Beckman Coulter, Pasadena, CA, USA), and plates were incubated for 72 h. The test compound was examined in triplicate with serial two-fold dilutions. After incubation, MTT was added and plates were gently shaken and incubated for 3 h at 37 °C in 5% CO_2_ incubator. The supernatant was removed and 100 mL of DMSO 100% was added. The plates were gently shaken to solubilize the originated formazan and absorbance at 570 nm was read in a Victor2 Wallac spectrofluorometer (Perkin Elmer, Waltham, MA, USA). IC50 values were calculated as the concentration that decreases 50% of the cell viability using Genedata Screener software (Genedata AG, Basel, Switzerland). Curve fitting followed the Smart Fit strategy with Hill model selection.

## 4. Conclusions

In summary, a new member of the lobophorin family that we have designated as lobophorin K has been obtained from cultures of *Streptomyces* sp. M-207, previously isolated from the cold-water coral *Lophelia pertusa* collected at 1800 m. Its structure was determined using a combination of spectroscopic techniques, mainly ESI-TOF MS and 1D and 2D NMR, and comparison with the NMR spectra of other members of the family. This new natural product displayed cytotoxic activity human pancreatic carcinoma (MiaPaca-2) and breast adenocarcinoma (MCF-7) human cells as well as moderate and selective antibiotic activity against pathogenic Gram-positive bacteria such as *Staphylococcus aureus.* The research described herein confirms once more that marine derived actinomycetes continue to be a rich and underexploited source of new small molecules that could lead to the discovery of new antibiotics and anticancer drugs.

## Figures and Tables

**Table 1:** ^1^H and ^13^C NMR (500 and 125 MHz in CD_3_OD) data for compound **1**.

Position	δ ^1^H (mult, *J*, Hz)	Δ ^13^C	Position	δ ^1^H (mult, *J*, Hz)	δ ^13^C
1	-	n.d.	-	-	-
2	-	100.7	A1	4.77 (br d, 4.1)	99.7
3	-	205.0 ^a^	A2	2.39 (m), 1.74 (m)	31.0
4	-	52.4	A3	4.02 (dd, 6.1, 2.9)	69.3
5	2.08 (m)	44.8	A4	3.26 (dd, 9.6, 3.3)	73.4
6	1.61 (m)	32.5	A5	4.10 (m)	66.0
7	1.61 (m), 1.53 (m)	43.0	A6	1.21 (d, 6.4)	18.2
8	2.24 (m)	35.9	-	-	-
9	3.42 (dd, 10.1, 5.3)	86.2	B1	5.17 (br d, 3.5)	93.2
10	2.09 (m)	39.7	B2	2.09 (m), 1.99 (dt, 14.6, 3.7)	35.9
11	5.81 (br d, 10.2)	127.2	B3	4.18 (dd, 6.5, 3.5)	68.1
12	5.38 (ddd, 10.2, 4.9, 1.8)	127.9	B4	3.29 (m)	83.3
13	3.65 (m)	52.7	B5	4.06 (m)	63.7
14	-	137.1	B6	1.21 (d, 6.3)	18.0
15	5.20 (brd, 9.4)	124.9	-	-	-
16	2.40 (m), 2.26 (m)	32.5	C1	4.95 (dd, 9.7, 1.7)	100.7
17	4.20 (m)	80.3	C2	2.05 (m), 1.72 (m)	38.8
18	-	139.3	C3	4.29 (dd, 5.8, 2.8)	64.4
19	5.12 (br d 10.5)	120.2	C4	2.85 (dd, 9.4, 2.8)	83.8
20	3.59 (br d, 10.4)	41.5	C5	3.81 (dq, 9.4, 6.2)	69.6
21	5.43 (br s)	122.7	C6	1.23 (d, 6.3)	18.6
22	-	142.5	C7	3.38 (s)	57.0
23	2.62 (m)	29.0	-	-	-
24	2.39 (m), 1.79 (d, 14.2)	36.4	D1	4.73 (dd, 9.8, 2.5)	99.2
25	-	84.9	D2	1.61 (m), 1.50 (dd, 14.3, 9.8)	37.5
26	-	200.9	D3	-	61.6
27	1.55 (s)	15.5	D4	3.66 (m)	54.1
28	0.65 (br d, 4.2)	22.9	D5	4.23 (qd, 6.4, 1.6)	69.4
29	1.14 (d, 7.0)	14.8	D6	1.08 (d, 6.4)	17.5
30	1.38 (br s)	14.2	D7	1.15 (s)	22.6
31	1.41 (br s)	15.3	D8	-	160.1
32	4.14 (br s), 4.09 (m)	65.0	D9	3.65 (s)	52.4
33	1.28 (d, 7.2)	20.5	-	-	-

n.d. = not detected; ^a^ detected via HMBC.

**Table 2:** MIC_90_ values of compound **1** against Gram-positive and Gram-negative bacteria.

Bacteria	Lobophorin K (MIC_90_ µg/mL)
Gram-positive	-
*Staphylococcus aureus* EPI1167 MSSA	40–80
*Staphylococcus aureus* MB5393 MRSA	>160
Gram-negative	-
*Acinetobacter baumannii* MB5973	>160
*Pseudomonas aeruginosa* PAO1	>160
*Klebsiella pneumoniae* ATCC 700603	>160
*Escherichia coli* MB2884	>160

## Supplementary Materials

The following are available online at www.mdpi.com/1660-3397/15/5/144/s1, Figure S1: UV spectrum of compound **1**; Figure S2: ESI-TOF spectra of compound **1**; Figure S3: ^1^H NMR spectrum (CD_3_OD, 500 MHz) of compound **1**; Figure S4: ^13^C NMR spectrum (CD_3_OD, 125 MHz) of compound **1**; Figure S5: COSY spectrum of compound **1**; Figure S6: HSQC spectrum of compound 1; Figure S7: HMBC spectrum of compound **1**; Figure S8: ROESY spectrum of compound **1**; Figure S9: Dose response curves of compound **1** against MCF-7, MiaPaca-2 and THLE-2 human cell lines.
