# Supplementary material for: Lobophorin K, a New Natural Product with Cytotoxic Activity Produced by Streptomyces sp. M-207 Associated with the Deep-Sea Coral Lophelia pertusa
Source: Mar Drugs. 2017 May 19;15(5):144. doi: 10.3390/md15050144 (PMC5450550; doi:10.3390/md15050144)
Supplement: Supplementary file 1 [file marinedrugs-15-00144-s001.pdf]

Supplementary Materials: Lobophorin K, A New Natural Product with Cytotoxic Activity Produced by Streptomyces sp. M-207 Associated with the Deep-Sea Coral Lophelia pertusa

Alfredo F. Braña, Aida Sarmiento-Vizcaíno, Miguel Osset, Ignacio Pérez-Victoria, Jesús Martín, Nuria de Pedro, Mercedes de la Cruz, Caridad Díaz, Francisca Vicente, Fernando Reyes, Luis A. García and Gloria Blanco

List of supplementary materials

Figure S1. UV spectrum of compound **1**.
Figure S2. ESI-TOF spectra of compound **1**.
Figure S3. ^{1}H NMR spectrum (CD_3OD, 500 MHz) of compound **1**.
Figure S4. ^{13}C NMR spectrum (CD_3OD, 125 MHz) of compound **1**.
Figure S5. COSY spectrum of compound **1**.
Figure S6. HSQC spectrum of compound **1**.
Figure S7. HMBC spectrum of compound **1**.
Figure S8. ROESY spectrum of compound **1**.
Figure S9. Dose response curves of compound **1** against MCF-7, MiaPaca_2 and THLE-2 human cell lines.

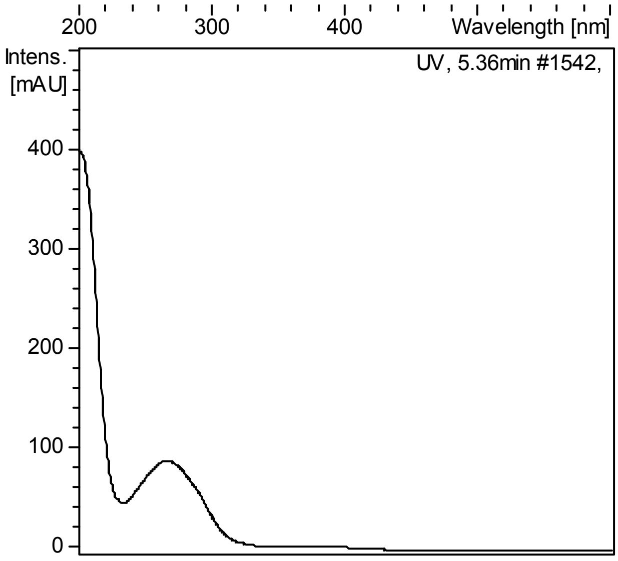

Figure S1. UV spectrum of compound **1**.

ISCID=100 eV

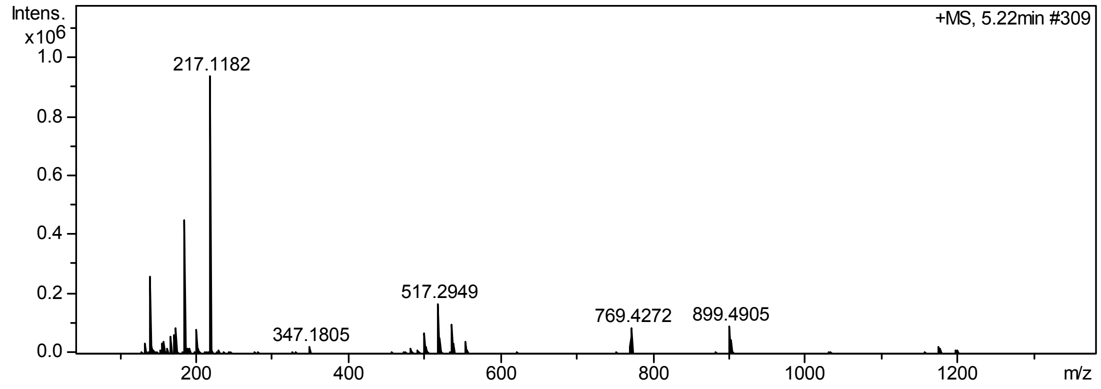

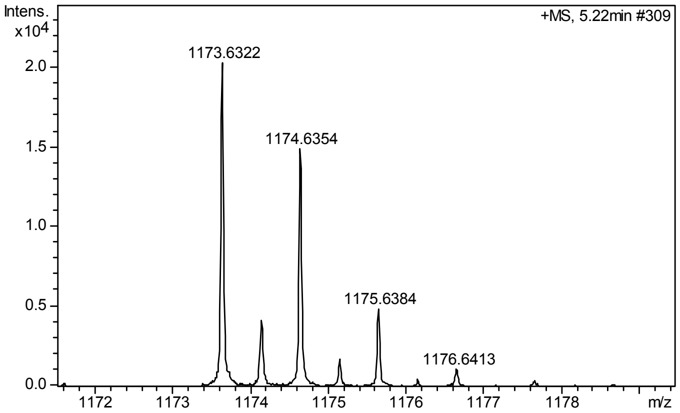

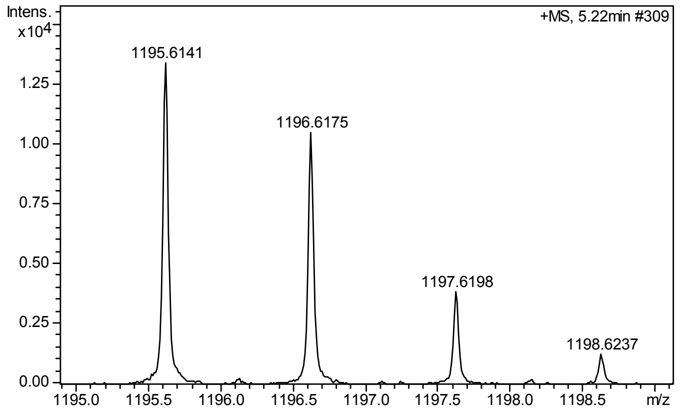

ISCID=0 eV

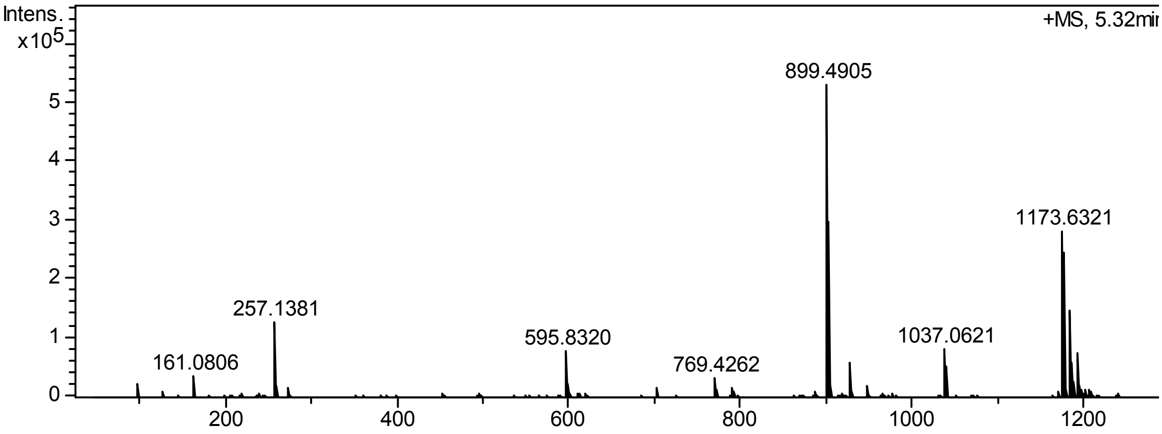

Figure S2. ESI TOF spectra of compound **1**.

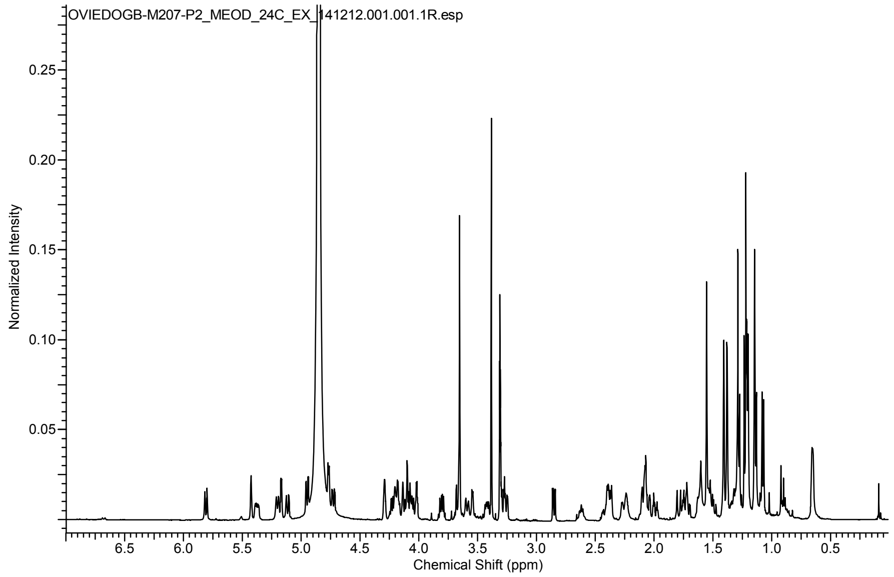

Figure S3. ^{1}H NMR (CD_3OD, 500 MHz) of compound **1**.

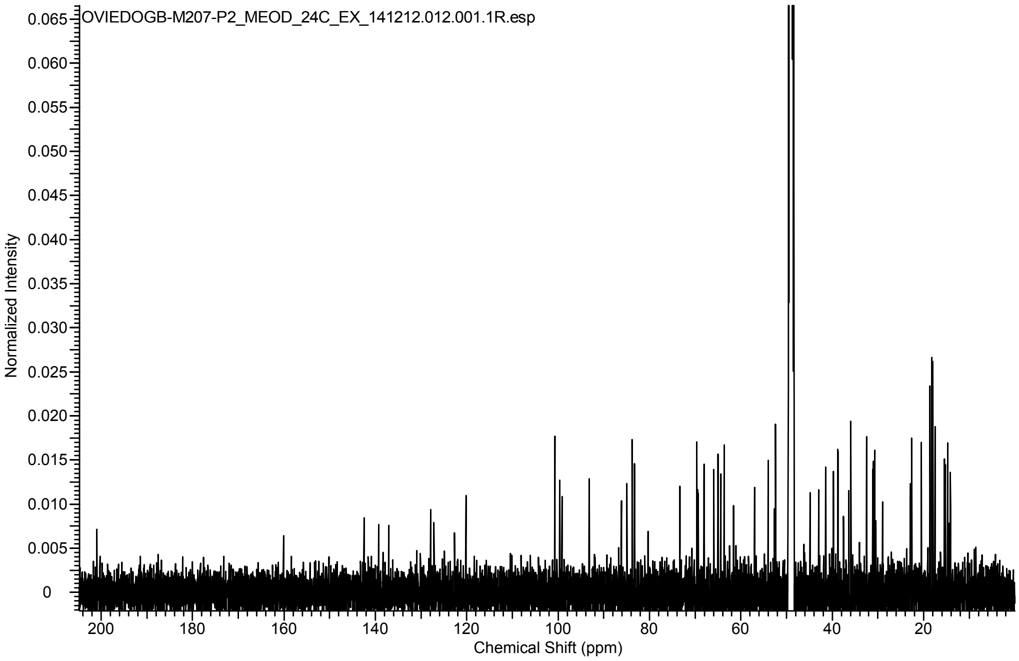

Figure S4. ^{13}C NMR (CD_3OD, 125 MHz) of compound **1**.

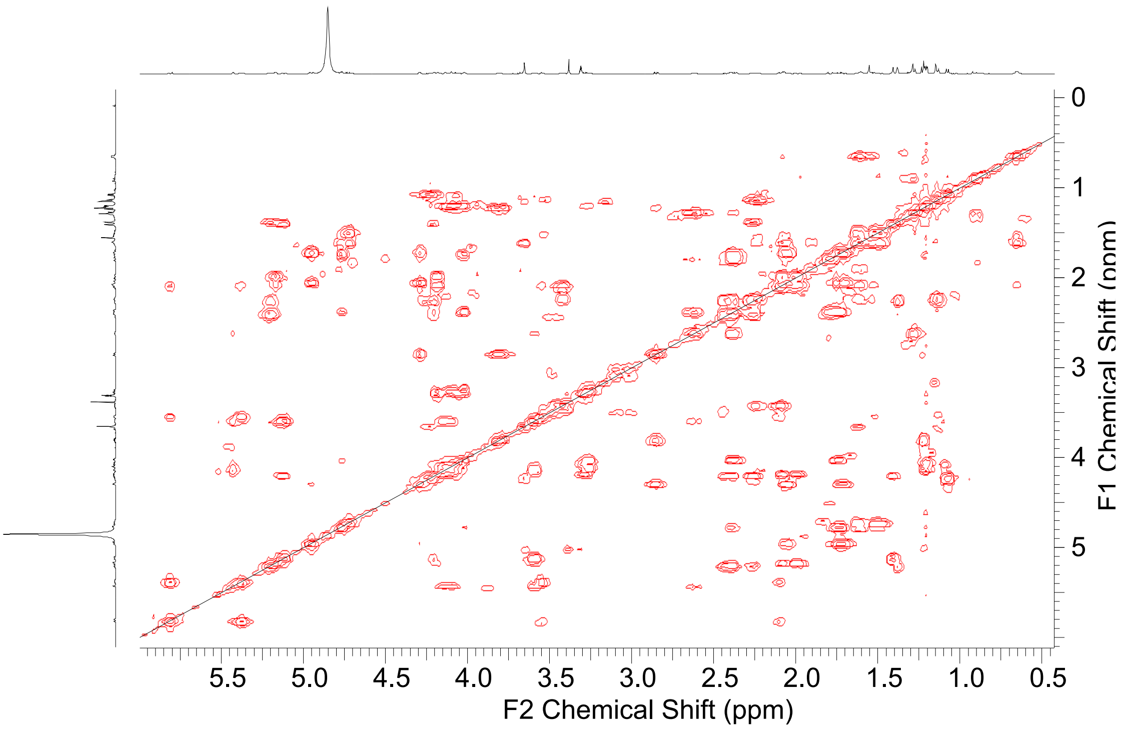

Figure S5. COSY spectrum of compound **1**.

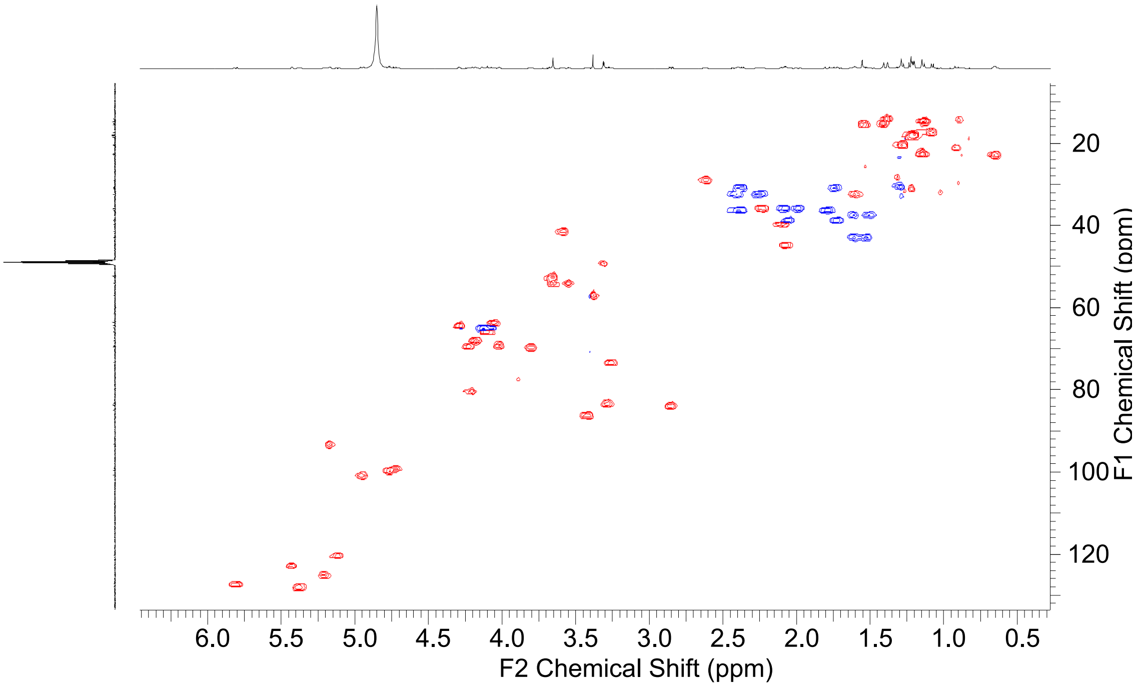

Figure S6. HSQC spectrum of compound **1**.

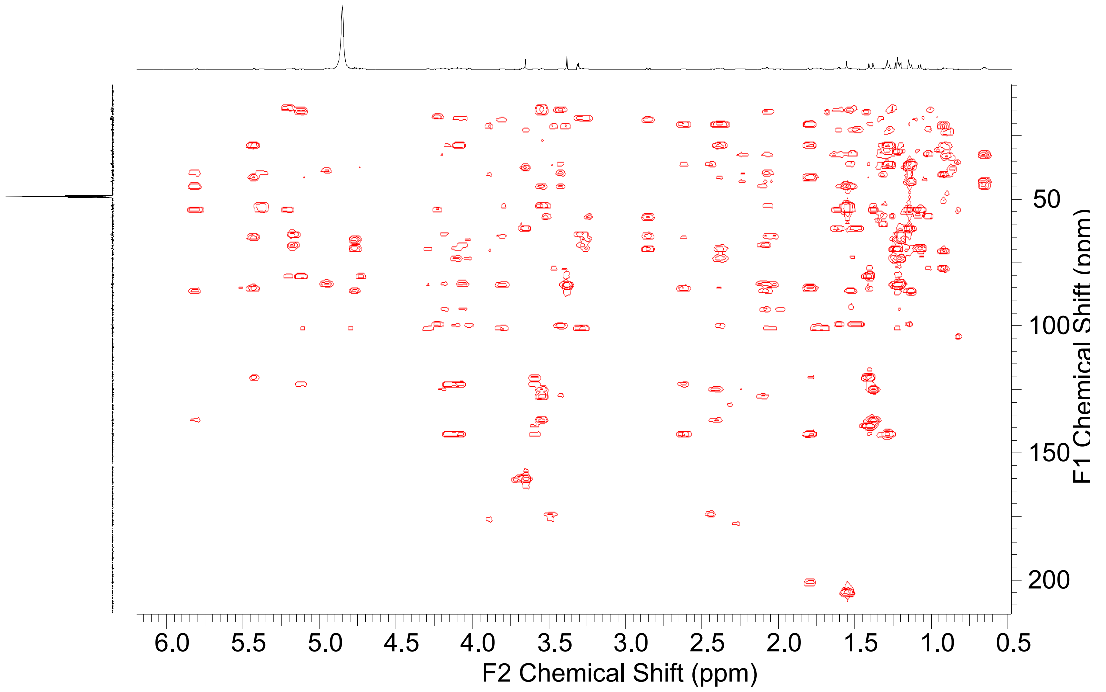

Figure S7. HMBC spectrum of compound **1**.

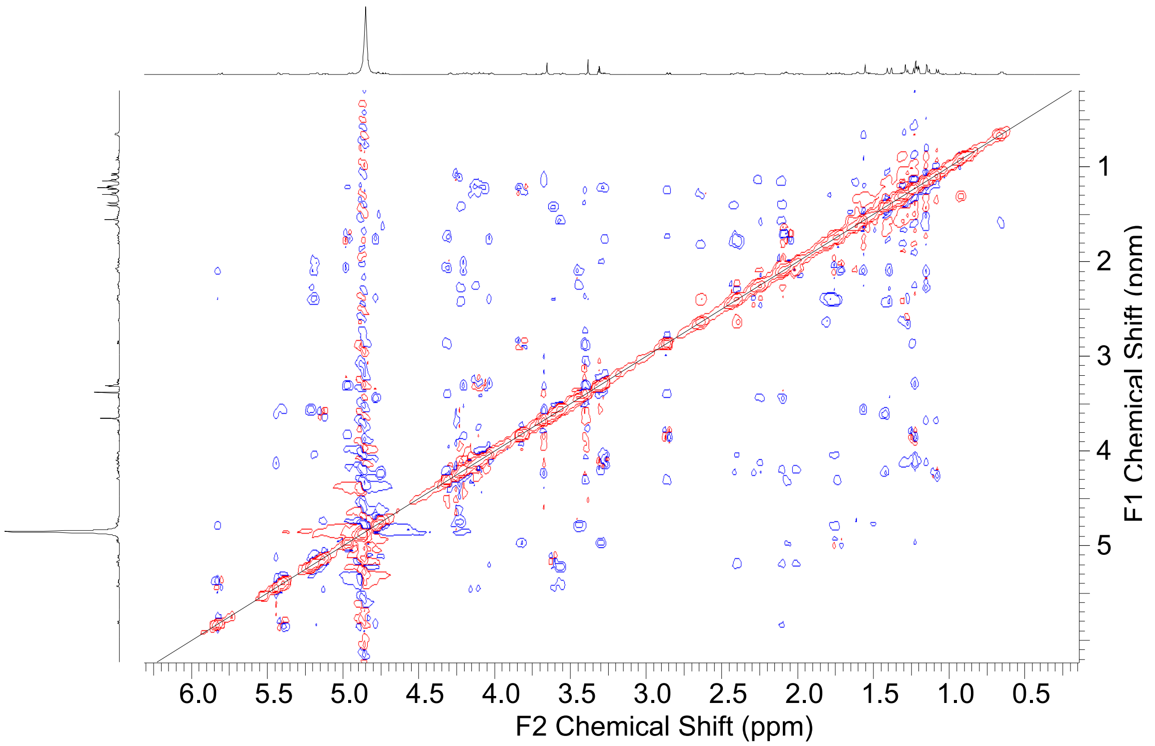

Figure S8. ROESY spectrum of compound.

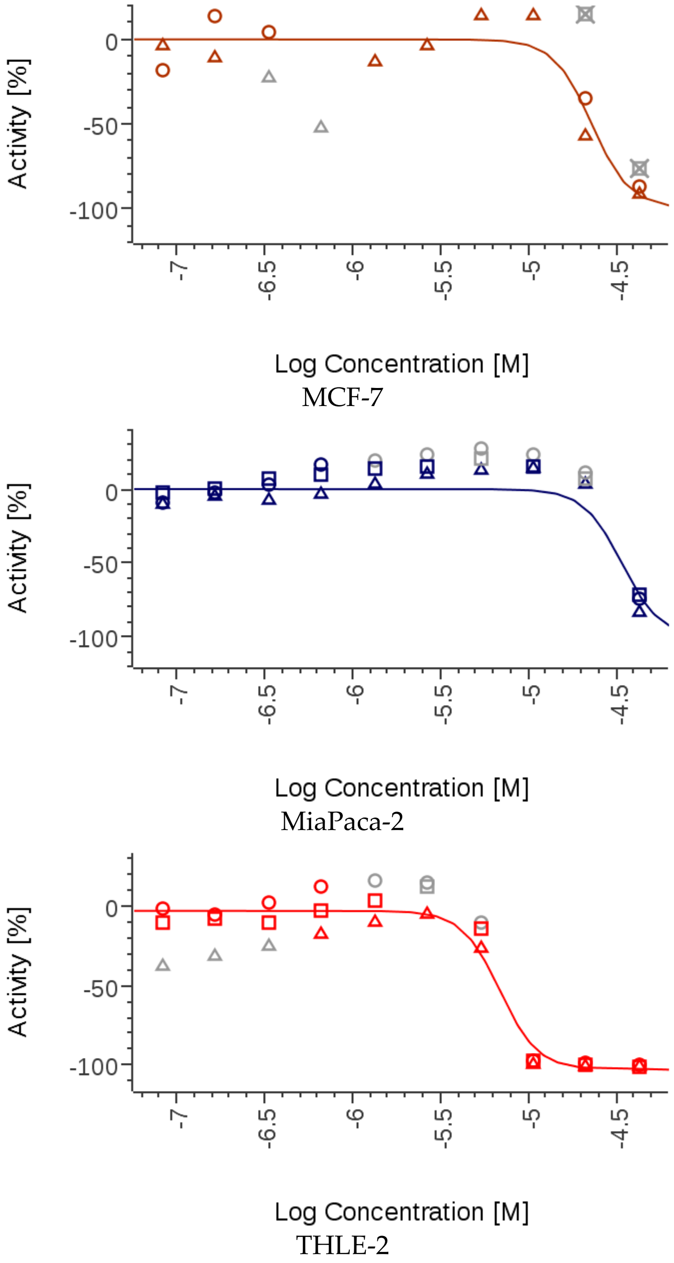

Figure S9. Dose response curves of compound **1** against MCF-7, MiaPaca_2 and THLE-2 human cell lines.
